# Criterion validity and reliability of a smartphone delivered sub-maximal fitness test for people with type 2 diabetes

**DOI:** 10.1186/s13102-016-0056-7

**Published:** 2016-10-07

**Authors:** Cecilie Fau Brinkløv, Ida Kær Thorsen, Kristian Karstoft, Charlotte Brøns, Laura Valentiner, Henning Langberg, Allan Arthur Vaag, Jens Steen Nielsen, Bente Klarlund Pedersen, Mathias Ried-Larsen

**Affiliations:** 1 Centre for Physical Activity Research, Finsen Centre, Rigshospitalet, University of Copenhagen, Blegdamsvej 9, 2100 Copenhagen, Denmark; 2 Department of Endocrinology (Diabetes and Metabolism), Rigshospitalet, University of Copenhagen, Blegdamsvej 9, 2100 Copenhagen, Denmark; 3 Department of Public Health, Section of Social Medicine, University of Copenhagen, Henrik Pontoppidans Vej 4, 2200 Copenhagen, Denmark; 4 Department of Endocrinology, Odense University Hospital, Sdr. Boulevard 29, Entrance 112, 3rd floor, 5000 Odense, Denmark; 5 The Danish Diabetes Academy, Odense University Hospital, Sdr. Boulevard 29, Entrance 112, 3rd floor, 5000 Odense, Denmark

**Keywords:** Fitness, Smartphone, Validity, Reliability, Type 2 diabetes mellitus, Exercise

## Abstract

**Background:**

Prevention of multi-morbidities following non-communicable diseases requires a systematic registration of adverse modifiable risk factors, including low physical fitness. The aim of the study was to establish criterion validity and reliability of a smartphone app (InterWalk) delivered fitness test in patients with type 2 diabetes.

**Methods:**

Patients with type 2 diabetes (*N =* 27, mean (SD) age 64.2 (5.9) years, BMI 30.0 (5.1) kg/m^2^, (30 % male)) completed a 7-min progressive walking protocol twice (with and without encouragement). VO_2_ during the test was assessed using indirect calorimetry and the acceleration (vector magnitude) from the smartphone was obtained. The vector magnitude was used to predict VO_2peak_ along with the co-variates weight, height and sex. The validity of the algorithm was tested when the smartphone was placed in the right pocket of the pants or jacket. The algorithm was validated using leave-one-out cross validation. Test-retest reliability was tested in a subset of participants (*N =* 10).

**Results:**

The overall VO_2peak_ prediction of the algorithm (R^2^) was 0.60 and 0.45 when the smartphone was placed in the pockets of the pants and jacket, respectively (*p <* 0.001). The mean bias (limits of agreement) in the cross validation was−0.4 (38) % (pants) and−0.1 (46) % (jacket). When the smartphone was placed in the jacket a significant intensity dependent bias (*r =* 0.5, *p =* 0.02) was observed. The test-retest intraclass correlations were 0.85 and 0.86 (*p <* 0.001), for the pants and jacket, respectively. No effects of encouragement were observed on test performance.

**Conclusion:**

In conclusion, the InterWalk Fitness Test is accurate and reliable for persons with type 2 diabetes when the smartphone is placed in the side pocket of the pants for. The test could give a fair estimate of the CRF in absence of a progressive maximal test during standardized conditions with the appropriate equipment.

**Trial registration:**

www.clinicaltrials.org (NCT02089477), first registered (prospectively) on March 14th 2014

## Background

Type 2 diabetes (T2D), along with a range of non-communicable diseases, has become an increasing societal burden [1–3]. Low cardiorespiratory fitness (CRF) is a marker of low health-related physical activity [4, 5] and an independent predictor of some of these non-communicable diseases including T2D and all-cause mortality [6–8]. Modifiable risk factors, such as low CRF, should be identified and targeted in order to implement strategies to prevent the development and consequences of these diseases. CRF is normally assessed in the laboratory by a comprehensive exercise test with expensive equipment, administered by trained test personnel and with the requirement of maximal effort from the participants. Hence, this procedure is not appropriate and feasible in populations selected for large scale testing. A number of field-applicable walking/running-based test protocols have been developed and tested [9]. Although criterion validity has been established for these protocols, they still require the presence of test personal, standardized surroundings, post processing and reporting of test results which makes them difficult to apply in large-scale testing and monitoring within clinical care and rehabilitation. Thus, novel low cost and valid methods to assess VO_2peak_ can improve the detection of people at risk and increase the use of VO_2peak_ as a risk stratification tool.

On-board movement sensors on smartphones allow for registration of exercise intensity. With the increasing use of smartphones, this platform might thus be a feasible tool for estimating CRF on a larger scale. Since new more feasible test for large scale testing and monitoring are needed we developed a smartphone delivered progressive walking CRF test for persons with T2D, as walking is considered safe and feasible, not least for the elderly population [10].

The aim of the study was to establish criterion validity and test-retest reliability of a 7-min progressive sub-maximal CRF test delivered by the smartphone app InterWalk. Secondarily, we aimed to examine the importance of the body location of the smartphone (the side pocket of the pants or jacket), and of therapist encouragement during the test on test performance.

## Methods

### Study sample

The present article describes a validation study that is a subset of a trial investigating the effect of SMS-prompting on the adherence to IWT (NCT02089477). Participants were recruited using bulletins. Potential participants contacted the project employees by telephone and were orally informed about the study. If no exclusion criteria (see below) were identified through the telephone interview, written material and consent form was mailed to the participant and participants was offered an information meeting. Interested participants screened by telephone was examined at a pre-examination and further informed about the study.

The inclusion criteria were; confirmed T2D diagnose (fasting glucose ≥7.0 mmol/L, random measured glucose ≥11.1 mmol/L, 2 h oral glucose tolerance test glucose ≥11,1 mmol/L or HbA1c > 48 mmol/mol), age˃30 years and BMI ˃18 but ˂40. Exclusion criteria included pregnancy, insulin dependence, contraindications to physical activity and any evidence of thyroid, liver, lung, heart or kidney disease [11]. Training status was not an in or exclusion criteria, however participant included were characterized with low physical fitness (VO_2max_/kg = 23,3 (±4,6) ml O_2_/kg/min) [12]. The sample for this validation study constitutes of participants with available data on VO_2peak_ and smartphone sample accelerometer data. No formal sample size calculation was therefore performed. The participants had either no or 3 months’ experience with the InterWalk app. Participants received oral and written information about the study and informed consent was obtained from all participants. The study is approved by the Regional Ethics Committee of the Capital Region of Denmark (H-1-2013-116).

### The Inter Walk app

The development of the InterWalk app and the background for development has been described in details elsewhere [13]. Briefly, the InterWalk app was developed as a vehicle to deliver interval walking training (IWT) for persons with T2D. The intensities during IWT are individualized based on the InterWalk Fitness Test (IWFT)-a standardized 7-min progressive walking test protocol (see below). During IWT and IWFT, on-board accelerometer data are sampled (100 Hz). The vector magnitude (VM) is calculated as the square root of the summed squared acceleration from the x, y and z axes. Subsequently, the data are averaged across 30 s. The data are transmitted to the server through Wi-fi or the mobile data network along with user demographics and central personal registration number [14].

### Procedures

Indirect calorimetry (described below) was used as the criterion measure to validate the prediction of VO_2peak_ from the accelerometer data obtained during the IWFT along with other co-variates. To establish test-retest reliability, the IWFT was repeated after 1 week in a subset of the participants (*N =* 10). Prior to testing, all participants underwent a medical screening including a health status interview and a physical exam, and demographic information was obtained.

### The Inter Walk Fitness test (IWFT)

The IWFT protocol consists of 2 minutes of slow, followed by 2 minutes of intermediate, 2 minutes fast and 1 minute of very fast walking. The walking intensities are self-selected, thus what “slow walking”, “fast walking” etc. means is defined by the user. The test protocol is audio guided through earphones. Hence, the app automatically instructs the user to start and change the walking intensities as described above.

After a thorough introduction to the test procedures and the InterWalk app, the participants completed two IWFTs during each visit (see below). The tests were administered by smartphones (Iphone 5C, IOS 7, v 1.18.8/2.0, Apple inc). All IWFTs were performed outside on a standardized course. The course was selected to reflect a free-living situation; i.e. the tests were performed on a side-walk near a major road (incl. traffic noise and other pedestrians) with turns and varying surface.

**Fig. 1:**
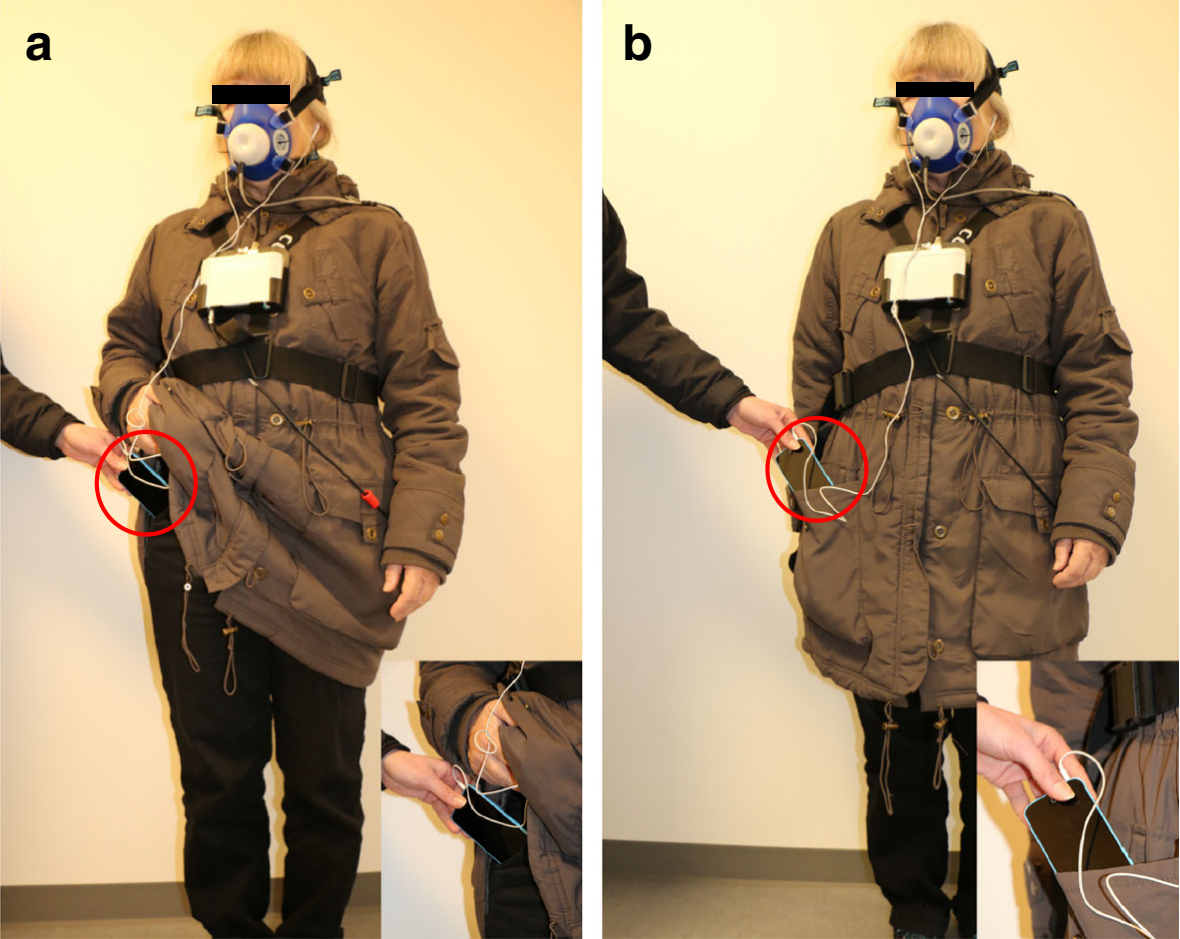
Photo of placements and setup. 1**a**: Smartphone placed in the side pocket of the pants (*lower position*, LP), 1**b**: Smartphone placed in the side pocket of the jacket (*upper position*, UP)

The first IWFT was self-administered with self-selected pace, performed only by the auditory instructions from the InterWalk app. The second test was conducted with self-selected pace by instruction from the InterWalk app along with verbal encouragement from the examiner to increase the subjects’ walking intensity during the last minute of the test. At both IWFTs the smartphones were placed in the right side pocket of the pants (lower position; LP) and in the right side pocket of the jacket (upper position; UP) (Fig. 1). A resting period of >20 min was employed between the two tests.

### Measurement of peak (VO_2peak_) and maximal oxygen consumption (VO_2max_)

VO_2peak_ was assessed during both IWTF procedures using indirect calorimetry (Cosmed K4b^2^, Cosmed, Italy) as indirect calorimetry is considered the gold standard in assessing VO_2_ in a clinical setting [15]. The device is a lightweight portable system calculating oxygen uptake from breath by breath measurements described in detail elsewhere [16] and has previously been validated [17]. The system was calibrated according to the manufacturer description. VO_2peak_ was defined as the mean oxygen consumption (ml O_2_) during the last 30 s of the protocol.

VO_2max_ was assessed by a graded walking test protocol on treadmill (Technogym Runrace, Gambettola, Italy). Since the majority of the participants don’t have the capacity to run, the walking test protocol was chosen. This test has previously been used successfully in our laboratory [18]. The participants were allowed to select their own comfortable walking speed ranging from 1.6 to 4.8 km/h at a 1 % incline during a 6-min warm-up. The warm-up was followed by 2 min intervals of increasing inclines (2 % per stage) at individually determined brisk walking, ranging from 2.2 to 5.5 km/h until two of the three following criteria were met: plateauing of VO_2_ with incremental workloads and/or respiratory exchange ratio˃1,0 and/or at exhaustion (as assessed by the examiner). Oxygen consumption was assessed using continuous indirect calorimetric measurements (CPET, Cosmed, Italy).

### Anthropometry

Height and weight were determined using standard procedures. Height was measured barefooted to the nearest 0.1 cm by a stadiometer (The Leichester Height Measure, Tanita). The weight was assessed during dual-energy X-ray absorptiometry scan (Lunar Prodigy Advance; GE Healthcare, Madison, WI).

### Prediction of VO_2peak_ during the IWFT

Tri-axial accelerometry has previously shown to predict energy expenditure (EE) during walking when the monitor was attached to the body [19–22]. Thus, the on-board accelerometer of the smartphone was used as predictor of VO_2peak_ during the IWFT. The remaining predictor variables were chosen *a priory*. As EE increases during walking with higher body weight and height, these parameters were included as co-variates in the prediction model along with sex [23]. We chose not to include other predictor variables due to consideration of feasibility, as the users of the InterWalk app provide this information during set up of the InterWalk app [13]. As the relationship between VO_2_ and acceleration has previously been shown to be linear during walking [19–22], we chose to build our prediction model using a linear regression model as described below;

1 $$ {Y}_i = {\beta}_0 + {\beta}_1{X}_{i1} + {\beta}_2{X}_{i2} + {\beta}_3{X}_{i3} + {\beta}_4{X}_{i4} + {\varepsilon}_i $$

Where Y_i_ is VO_2peak_ (ml/min) during the last 30 s. of the test, X_i1_ is the mean VM (g) during the last 30 s of the test, X_i2_ is the body weight, X_i3_ is the body height (cm), X_i4_ is a sex indicator (0 for women, 1 for men) and ε_i_ is the error term. Standard linear regression diagnostics, including examining linearity, homoscedasticity, multi-collinearity and normal distribution of the residuals were performed. No indications of violations of the linear regression assumptions of the prediction equations were observed.

### Statistical analyses

To established criterion validity we correlated the product–moment correlation coefficient (r) between the VM and oxygen consumption [24]. In order to obtain an un-biased estimate of the precision and accuracy of the predicted VO_2peak_, we calculated the predicted VO_2peak_ using the leave-one-out cross validation (LOOCV). Agreement between the predicted values from the LOOCV and the observed values are evaluated using Bland-Altman plots with mean bias and limits of agreement (LOA) [25].

Reproducibility (test-rest) was expressed as the intraclass correlation coefficient (ICC) of the predicted values across 1 week. The interpretation of the reproducibility was based on the size of the ICC and classified as good to excellent (1.00–0.76), fair to good (0.75–0.41) and poor (0.41–0.00) [26]. Furthermore, we calculated the minimal detectable change (MDC) not due to measurement error of the predicted VO_2peak_. The MDC was calculated as 1.96*√2*(Standard deviation_MD (1week follow-up and baseline)_/√2) [27, 28]. All analyses were performed using STATA IC 13.1 (Stata Corp, Texas, USA). Statistical significance was set at α˂ 0,05 (two tailed).

## Results

### Sample characteristics

A total of 27 participants (31 % with previous InterWalk app experience) completed the protocol. Sample characteristics are described in Table 1. Participants with and without previous experience with the InterWalk app did not differ in VO_2max_ and VO_2peak_. However, participants with previous experience were heavier (MD [95 % CI]) (12.1 kg [1.0;23.1]), had a higher BMI (4.1 [0.5;7.5]) kg/m^2^ and a higher HbA1c (ratio of geometric mean [95 % CI] 1.11 [1.08;1.16]). The participants reached (mean [95 % CI]) 85 % [79.0 to 90.0] of their VO_2max_ during the IWFT.

**Table 1:** Sample characteristics

	Total	Men	Women
*N*	27	9	18
*Age (years)*	64.2 (5.9)	65.9 (6.8)	63.3 (5.5)
*Level (no exp./previous exp.)*	16/11	5/4	11/7
*Time since T2D diagnosis*	7.9 (5.0)	8.3 (5.3)	7.6 (4.9)
*Body weight (kg)*	83.2 (14.9)*	90.0 (13.4)	79.7 (14.7)
*Body height (cm)*	166.6 (7.7)*	175.2 (4.0)	162.2 (4.7)
*Body mass index (kg/m* ^*2*^ *)*	30.0 (5.1)	29.3 (4.9)	30.3 (5.3)
*HbA1c (mmol/mol)*	47.0 [41.0;57.0]	49.0 [45.0; 57.0]	47.0 [38.0;50.0]
*HbA1c (%)*	6.4 [5.9; 7.4]	6.6 [6.3; 7.4]	6.4 [5.6; 6.7]
*Anti-diabetic medication (yes/no))*	23/4	6/2	16/2
*VO*_*2max*_ *(ml/min)*	1940 (457)*	2314 (427)	1773 (369)
*Relative VO*_*2max*_ *(ml/kg/min)*	23.3 (4.6)	25.3 (4.6)	22.5 (3.9)

Data are mean (standard deviation) or median [Inter quartile range], HbA1c; Glycated hemoglobin A1c. Sex differences (* *p <* 0.05) were tested using student’s *t*-test for normally distributed variables and Wilcoxson’s rank sum test for non-normally distributed variables

### Effects of encouragement and placement of the smartphone on VO_2peak_ and vector magnitude

Table 2 describes the observed VO_2peak_ and VM during the last 30 s of the 7-min IWFT. No difference in VO_2peak_ was observed when the test was completed with encouragement compared with completion without encouragement (*p =* 0.70). Nor did the VM differ between tests (*p =* 0.40 and 0.12 for differences between encouragement and no encouragement, when the smartphone was placed in the LP and UP, respectively). However, a lower VM was observed when the smartphone was placed in the UP, compared to the LP, when the test was performed without (MD [95 % CI]) (−0.17 G [−0.22;−0.11]) as compared to encouragement (MD [95 % CI]) (−0.18 G [−0.23;−0.12]).

**Table 2:** VO_2peak_ and vector magnitude during the last 30 s of the walking test

	VO_2peak_(ml/min)	Vector magnitude (LP) (G)	Vector magnitude (UP) (G)
N	27	27	27
No encouragement	1602 (427)	0.30 [0.22;0.46]	0.17 [0.14;0.20]*
Encouragement	1651 (436)	0.39 [0.25;0.51]	0.19 **[**0.14;0.24]*

Data are means (standard deviation) or medians (interquartile range). *p < 0.001 for differences between body positions, LP; lower position, smartphone placement in the right side pocket of the pants, UP; Upper position, smartphone placement in the right side pocket of the jacket. Differences were tested using Wilcoxon’s matched-pair signed-rank test

### Accuracy and precision of the predicted VO_2peak_

**Fig. 2:**
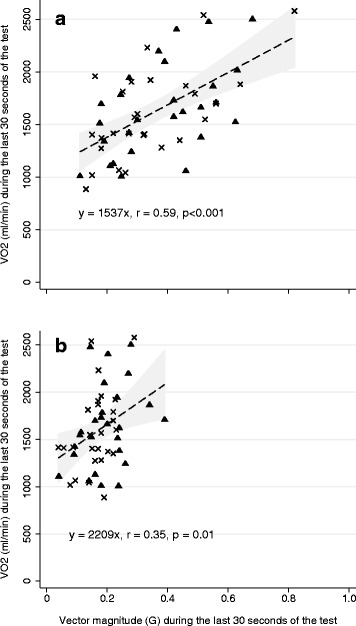
The partial correlations between VO_2peak_ and vector magnitude during the last 30 s of the test when the smartphone was placed in the lower position, smartphone placement in the right side pocket of the pants (Panel **a**) and in upper position, smartphone placement in the right side pocket of the jacket (Panel **b**). The dotted line is the best fit line. Triangles (▲) represent observations during the test with encouragement and crosses (X) are observations during the test without encouragement. The grey shaded area is the 95 % confidence interval

The correlations between VO_2peak_ and VM during the last 30 s of the test for the LP and UP are depicted in Fig. 2. Data from the tests with and without encouragement were pooled as no differences between the r-values were observed between the LP (r_encouragement_ [95 % CI] = 0.63 [0.33;0.82], r_no encouragement_ [95 % CI] = 0.54 [0.20;0.76]) or the UP (r_encouragement_ [95 % CI] = 0.31 [−0.08;0.63], r_no encouragement_ [95 % CI] = 0.42 [0.05;0.70]). To account for the repeated measurements in the pooled analysis, we adjusted the standard errors for within-participant clustering (using *VCE cluster option*, Stata IC 13). The regression equation for prediction of VO_2peak_ during the last 30 s. of the 7-min IWFT for the LP was described as;

2 $$ V{O}_{2 peak}=1853*VM\ (G) + 11.8*\  weight\ (kg) + 25.2* height\ (cm)\ \hbox{--}\ 615.5* sex\ \left(0\ for\  women,\ 1\ for\  men\right)\ \hbox{--}\ 4006\ \left[2\right],\ {r}^2 = 0.60,\ p<0.001 $$

And for the UP as;

3 $$ V{O}_{2 peak}=2379*VM\ (G) + 15.0*\  weight\ (kg) + 11.3* height\ (cm)\ \hbox{--}\ 77.8* sex\ \left(0\ for\  women,\ 1\ for\  men\right)\ \hbox{--}\ 1901,\ {r}^2 = 0.45,\ p<0.001 $$

**Fig. 3:**
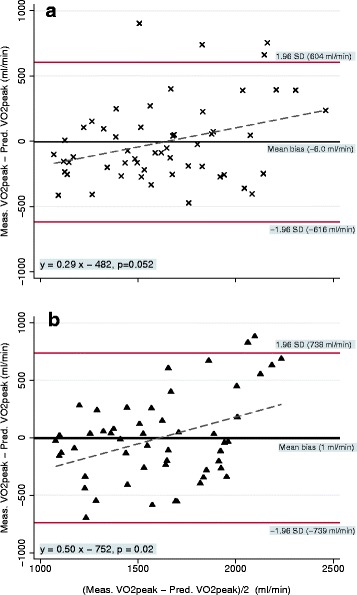
The accuracy (mean bias) and precision (±1.96 Standard deviation (SD)) of the VO_2peak_ prediction algorithms for the lower (panel **a**) and the upper (panel **b**) position of the smartphone placement in the ‘leave-on-out cross validation’. The dotted line is the best fit line and represents the bias of the predicted values. Triangles (▲) represent the predicted values from the VM derived from the smartphone when placed in the upper position and crosses (X) are predicted values from the lower position

Figure 3 describes the accuracy and precision of the predictions algorithms for the LP (Fig. 3a) and for the UP (Fig. 3b) from the leave-one-out cross validation. The precision was [LOA ± 46.8 %] for the UP and [LOA ± 35.5 %] for the LP.

### Reliability and the minimum detectable difference of the predicted VO_2peak_

The reliability (ICC [95 % CI)]) was 0.86 [0.64; 0.96] of the predicted VO_2peak_ for the LP. For the UP the reliability (ICC [95 % CI)]) was 0.85 [0.60; 0.96]. The MDC was 298 ml O_2_ for the LP and 203 ml O_2_ for the UP.

### Prediction of VO_2max_

The predicted VO_2peak_ correlated with the measured VO_2max_ (r_L*P =*_ 0.70, *p <* 0.001 and r_U*P =*_ 0.56, *p =* 0.004). The predicted VO_2peak_ underestimated the VO_2max_ by 15.5 % (*p <* 0.001) for the LP and 14.2 % (*p <* 0.001) for the UP. The mean bias [LOA] was 308 [215 to 401] ml O_2_ for the LP and 272 [118 to 427] ml O_2_ for the UP. The equations for both positions (LP and UP) underestimated the VO_2max_ for participants with higher VO_2max_ values and underestimated for the participants with lower values. The correlation between the bias and the mean of the VO_2max_ and the VO_2peak_ values was *r =* 0.36 (*p <* 0.01) for the LP and *r =* 0.46 (*p <* 0.02) for the UP.

## Discussion

The aim of this study was to present a VO_2peak_ protocol delivered by a smartphone app (InterWalk) and to establish criterion validity and test-retest reliability of a 7-min progressive fitness test. In addition we examined whether validity was influenced by the body location of the smartphone and if the test performance was affected by therapist encouragement. The accuracy and precision of the predicted VO_2peak_ were dependent on body location, i.e. higher when the smartphone was placed in the LP. Furthermore, encouragement during the last minute of the test did not seem to affect test performance in this sample.

### Differences between body locations

The VO_2peak_ was predicted with a high accuracy for both body locations (LP and UP) along with comparable reliability. However, when the smartphone was placed in the UP, the VO_2peak_ was overestimated at the higher intensities and underestimated at the lower intensities and the LOAs were wider. The partial correlation coefficients revealed that the algorithm was less dependent on the only dynamic test parameter in the algorithm (VM) for the UP compared to the LP and the recorded acceleration was generally lower. This would make the prediction algorithm less sensitive to variation herein. Differences in the validity for predicting VO_2_ between the placements of the accelerometer-based physical activity monitors are consistent with previous studies [29–31]. When the smartphone was placed in the UP, the accelerometer is closer to the centre of mass as observed when accelerometers are fixed on the hip or lower back. In contrast to the use of research physical activity monitors, the type of jackets, worn by the participants in the present study, was not standardized. Thus, some participants wore loosely fitted jackets and others wore jackets more closely attached to the body, all with different size pockets. The lower accuracy and higher variation of the IWFT when the smartphone was placed in the UP, might be due to movements of the jacket not corresponding to the actual movement of the body and therefore induce measurement error. When the smartphone was placed in the LP the accelerometer where close to the thigh/hip following the movement of the leg during walking closely and thus, the measurement error would be lower, explaining the narrower LOA for the LP. As the acceleration is the only body movement intensity-dependent variable in the equation, it could be speculated that the underestimation at higher VO_2peak_ values is due to low sensitivity to capture the body acceleration. If increases in the observed acceleration on the smartphone either do not reflect or underestimate the concomitant acceleration in body, i.e. centre of mass, an underestimation would be observed at higher intensities. However, we did not measure the actual acceleration of the body during this study. The acceleration signal was un-filtered, i.e. all movement frequencies of the smartphone were included. The VM used in the IWFT was calculated as an average across a 30 s. epoch, and thus would suppress high frequency noise components to some extent. It is possible that high and low frequency acceleration components, not related to body movement per se, could explain the low precision of the estimates across both body locations.

Several field-based walking tests have been developed and validated. In a recent meta-analysis Mayorga-Vega et al. (2016) reported low-to-moderate (*r =* 0.42–0.79) criterion-related validity for walk/run field tests to estimate VO_2max_ across 123 studies [9]. This is comparable to the IWFT, when the smartphone was placed in the LP. However, the studies reported on did not include patients. When compared to the criterion validity for the commonly used six minute walking test (6MWT) in patient groups characterized with low VO_2peak_, the criterion-related validity was slightly better for the IWFT [32, 33].

### Clinical feasibility

The performance during field-based walking tests may be affected by encouragement from test personnel, increasing the resources needed to implement them in clinical care. [33, 34]. Differences in walking distance have been observed in elderly people [32, 33, 35] and in patients with pulmonary limitations [36, 37] when the 6MWT is performed repeatedly. No differences in VO_2peak_ were observed, neither when the IWFT was performed self-conducted, nor with encouragement during the test. In view of the similar performance with and without encouragement and the high reproducibility (ICC; LP 0.86 and UP 0.85), the IWFT can be performed without the presence of professional test personnel, which will increase feasibility of the test in clinical and rehabilitation programs. Moreover, the high reliability indicates that the test has a very high consistency across from one test administration to the next [38], making it feasible to implement. The MDC not due to measurement error indicates the sensibility of the measurement to detect a change in the parameter of interest. Thus, the MDC indicates that the IWFT is able to detect changes in VO_2_ larger than ±18 % and ±13 % for the LP and UP, respectively. The exercise-induced magnitude of improvements in fitness level is related to the initial fitness level. Thus, only small changes (~5%) in CRF are observed in well trained individual (VO_2max_ ≥ 3500 ml O_2_/min) with training programs of high intensity [39, 40]. In contrast high intensity exercise interventions in T2D patients with low initial VO_2max_ have shown larger improvements in CRF. For example, improvements in CRF of 16-25 % were observed after 5 months of IWT [18, 41] and improvements of the same magnitude (18–46 %) were demonstrated in other patient groups undergoing high intensity exercise interventions [42, 43]. Therefore, the IWFT could be used as a tool to evaluate these types of exercise programs.

We performed a *post hoc* analysis to assess the relative validity [38]. The sensitivity and specificity of the predicted VO_2peak_ values to classify the participants into low/intermediate and high risk as compared to the observed VO_2peak_ values were thus calculated. The cut off values were based on reference values published by Kodoma et al. [8]. High risk participants were characterized with a VO_2peak_ <7.9 metabolic equivalents. The sensitivity was 98 % and 94 % for the LP and UP, respectively. The specificity was 77 % for both positions. Thus, to stratify individuals at risk, the post hoc analysis indicated a similar performance across body locations. However, the sample was very homogenous and characterized by poor fitness as compared to the normal population [12]. In view of the small sample size, the interpretation of the relative validity should thus be done with caution.

### Strengths and limitations

The main strengths of the study include the direct measurement of VO_2peak_, the well-characterized sample and an objective indirect measure of intensity. The validity and reliability of the IWFT were furthermore tested in a non-laboratory condition, increasing feasibility of usage in clinical care and rehabilitation without access to a laboratory. Finally, the independence of therapist encouragement on test performance enables patients to perform the test by themselves. With the online data upload the patients’ performance can be monitored over the distance, enabling use in large scale health surveillance programs as well as decreasing the workload of clinicians and therapists.

Some limitations to the study need to be addressed. First, the homogeneity of the sample and the relatively small sample size could limit the generalizability of the findings. However, the cross-validation demonstrated high accuracy and reliability of the equations. Therefore, we are confident that the equations can be employed in other populations with similar characteristics without loss of accuracy. Furthermore, we did not base the sample size on a formal sample size calculation, thus the study might have been under powered to detect statistically significant differences between the predicted and measured VO_2peak_ values. However, the difference was very low (<10 ml O_2_*min^−1^), and within the measurement error of the criterion measure Cosmed K4b^2^ (Cosmed, Italy) [16]. Second, the estimation of VO_2_ with accelerometers used during incline walking is not accurate [44]. The IWFT was performed on level surface, thus limiting the performance of the equations to surfaces without incline. Third, many studies do indeed observe an association between e.g. VO_2max_ rather than VO_2peak_ [8] as predicted by our equations. However, this association is still present when using VO_2peak_ as a predictor [8]. Fourth, due to the short resting period (20 min) between the paced and non-paced protocol and the lack of a randomized order of the administration of the protocol, fatigue could have prohibited the participants to reach a higher VO_2peak_ during the paced protocol. However, no difference in the RER-values was observed (mean difference [95 % CI] 0.02 [−0.03; 0.07], *p =* 0.44 between the paced and the non-paced protocols). Thus, we do not believe that the resting period and the lack of randomization explains the lack of effect of pacing the participants during the protocol. Fifth, the IWFT is a measure of VO_2peak_ during walking and thus might not be feasible for exercise prescription for other exercise modalities.

## Conclusion

In conclusion the IWFT is a valid and reliable tool in estimating VO_2peak_ in persons with similar characteristics as this sample, i.e. patients with a low VO_2max_, during walking. The IWFT displays similar criterion validity as other commonly used field-based walking tests. In order to obtain the highest accuracy and precision, the smartphone should be placed in the side pocket of the pants. The fact that no effect of encouragement on test performance was observed with high reliability indicates that the IWFT is feasible for self-administration as well as for use in the clinic. With the automatic transmission of test results, the IWFT makes it possible to test large groups of people over distance in a time-saving and economical manner. The test could give a fair estimate of the CRF in absence of a progressive maximal test during standardized conditions with the appropriate equipment.

## Abbreviations

6MWT: 6-min walking test
BMI: Body mass index
CI: Confidence interval
Cm: Centimeter
CRF: Cardio respiratory fitness
EE: Energy expenditure
HbA1c: Glycolated haemoglobin 1 ac
ICC: Intra class correlation
IWFT: InterWalk fitness test
IWT: Interval walking training
Kg: Kilogram
LOA: Limits of agreement
LOOCV: Leave one out cross validation
LP: Lower position
MD: Mean difference
MDC: Minimal detectable difference
T2D: Type 2 diabetes
UP: Upper position
VM: Vector magnitude
